# Bicarbonate ringer’s solution could improve the intraoperative acid-base equilibrium and reduce hepatocellular enzyme levels after deceased donor liver transplantation: a randomized controlled study

**DOI:** 10.1186/s12871-023-02383-8

**Published:** 2023-12-19

**Authors:** Qingkai Li, Ying Liu, Yanan Wang, Xin Shan, Chunxiao Liu, Zhihua Li, Jinglin Cao, Jian Dou, Guanjie Xu, Qiujun Wang, Xiaojuan Qie

**Affiliations:** 1 Department of Aesthesiology, The Third Hospital of Hebei Medical University, Shi Jiazhuang, 050051 China; 2 Department of Hepatobiliary Surgery, The Third Hospital of Hebei Medical University, Shi Jiazhuang, 050051 China

**Keywords:** Liver transplantation, Crystalloid, Bicarbonate Ringer’s solution, Acid-base equilibrium, Liver function

## Abstract

**Background:**

Bicarbonate Ringer’s (BR) solution is a direct liver and kidney metabolism-independent HCO_3_^−^ buffering system. We hypothesized that BR solution would be more effective in improving acid-base equilibrium and more conducive to better liver function than Acetate Ringer’s (AR) solution in conventional orthotopic liver transplantation (OLT) patients.

**Methods:**

Sixty-nine adult patients underwent OLT. Patients in the bicarbonate and acetate groups received BR solution or AR solution as infused crystalloids and graft washing solution, respectively. The primary outcome was the effect on pH and base excess (BE) levels. The secondary outcome measures were the incidence and volume of intraoperative 5% sodium bicarbonate infusion and laboratory indicates of liver and kidney function.

**Results:**

The pH and absolute BE values changed significantly during the anhepatic phase and immediately after transplanted liver reperfusion in the bicarbonate group compared with the acetate group (all *P* < 0.05). The incidence and volume of 5% sodium bicarbonate infusion were lower in the bicarbonate group than in the acetate group (all *P* < 0.05). The aspartate transaminase (AST) level at 7 postoperative days and the creatine level at 30 postoperative days were significantly higher in the acetate group than in the bicarbonate group (all *P* < 0.05).

**Conclusion:**

Compared with AR solution, BR solution was associated with improved intraoperative acid-base balance and potentially protected early postoperative liver graft function and reduced late-postoperative renal injury.

## Introduction

Conventional orthotopic liver transplantation (OLT) is complicated and involves a high risk of acid-base imbalance and drastic haemodynamic fluctuations [1]. Heming [2] et al. emphasized that the intravenous infusion of crystalloids is a key process to ensure tissue perfusion, cell oxygenation and physiological status in OLT patients. In addition, using a physiological solution such as a crystalloid solution to flush out preservation fluid could eliminate harmful metabolites inside the donor liver in both conventional OLT and piggyback liver transplantation (LT), and retrograde perfusion (reperfusion through the inferior vena cava (IVC), which is normally the main outflow channel) could reduce the risk of acid-base imbalance as well as drastic haemodynamic fluctuations after recirculation [3, 4]. The choice of perioperative crystalloid is a key influencing factor for acid-base balance, electrolyte disturbances, and important organ function, such as kidney, intestine and liver function [5].

Although several improvements in crystalloids have been made in the past decade, more effective crystalloids for OLT are lacking. Recent evidence suggests that acetate-buffered solutions result in better hemodynamic stabilization than 0.9% saline in patients undergoing major surgery and also reduce lactate levels compared with LR in infants with biliary atresia [6, 7]. Compared with saline, the infusion of AR solutions during OLT can reduce the occurrence of hyperchloremic acidosis and reduce the risk of acute kidney injury [8].

However, due to poor liver function and the anhepatic phase in LT patients, the liver has poor metabolic capacity for acetic acid. Lv [9] et al. criticized the use of AR solution as ineffective in improving microcirculation and alleviating acidosis. The administration of AR solution might lead to increased liver metabolic burden and even affect the early function of the transplanted liver [10].

To overcome these problems, a new type of crystalloid solution, bicarbonate Ringer’s (BR) solution has recently been used for fluid resuscitation. Compared to AR solution, BR solution is characterized as a direct liver and kidney metabolism-independent HCO^3−^ buffering system; therefore, it can more rapidly buffer acid and maintain acid-base balance without increasing the oxygen demand and hepatic burden [11]. Furthermore, Wang [12] et al. reported that BR solution could relieve ischaemia‒reperfusion injury (IRI) in liver cells.

However, whether BR solution is more effective in acid-base equilibrium and more conducive to better liver function than AR solution remains unclear. To date, and to our knowledge, there has been a paucity of evidence in the literature on the comparative influence of AR or BR solutions. Therefore, our primary objective in this randomized controlled trial was to compare the effects of AR or BR solutions infused during surgery and used to prepare the 2% albumin solution for graft washing on pH and base excess (BE) levels in OLT patients. The secondary outcome measures were intraoperative 5% sodium bicarbonate infusion incidence and volume, as well as laboratory indicators related to liver and kidney function.

## Methods

### Study design

The study was designed as a prospective, single-centre, double-blinded, randomized controlled trial that follows the ethical standards stated in the Declaration of Helsinki and the Consolidated Standards of Reporting Trials (CONSORT) reporting guidelines. It was approved by the Medical Ethics Committee of the Third Hospital of Hebei Medical University (Approval No. 2021-003-1) and registered at ClinicalTrials.gov (ChiCTR2100050486; principal investigator: Xiaojuan Qie, M.D.; 28/08/2021). All participants provided written informed consent before enrolment.

### Sample size

On the basis of the results of a previous study [13], we assumed that the difference between the groups with respect to the primary outcome of BE would be 0.31 mmol/L with a standard deviation of sample difference of 0.12, and thus, 32 patients were required in each group to achieve the desired power of 90% (β = 0.10) at the 5% (α = 0.05) level of significance.

### Participants

The study was conducted in the Department of Anaesthesiology of the Third Hospital of Hebei Medical University. From August 2021 to December 2022, adult patients (> 18 years) undergoing OLT under general anaesthesia were recruited. We excluded patients who had ASA scores of 5, patients who needed preoperative renal replacement therapy (RRT), patients who had a history of myocardial infarction within 6 months, patients who had hypermagnesemia or hypothyroidism, and patients who refused to participate in clinical trials or participate in other clinical studies within 3 months.

### Randomization and blinding

Patients were randomly assigned to receive AR or BR solution at a 1:1 ratio. The patients, anaesthetists and outcome adjudicators were blinded to the intervention assignment. Patients were assessed by a single investigator, and eligible patients were enrolled. When the patients entered the operating room, the second researcher opened a sealed envelope and carried out infusion and lavage according to the grouping. The anaesthesiologists were blinded to the allocated intervention by sterile curtain separation. Two other researchers collected the experimental data.

### Anaesthesia management and surgery

In our centre, anaesthesia for liver transplantation is accomplished via a standard protocol; all patients were monitored first by electrocardiogram, noninvasive blood pressure monitoring, and pulse oximetry. Radial artery puncture catheterization was performed under local anaesthesia, and the FloTrac/Vigileo monitoring system (Edwards Lifescience, USA) was connected to continuously monitor cardiac output (CO), cardiac index (CI), and stroke volume variability (SVV). The A-2000 BIS monitor (Aspect Medical System, USA) was used to monitor BIS values. After anaesthesia induction, when the patient’s BIS value reached 50 ~ 60 and the muscle relaxation was satisfactory, endotracheal intubation was performed for mechanical ventilation. A central venous pressure (CVP) catheter was inserted into the right internal jugular vein under ultrasound guidance.

Intraoperative anaesthesia was maintained with sevoflurane, propofol and remifentanil. Goal-directed fluid management established on SVV was used to guide fluid infusion. From the beginning of anaesthetic induction, the participants were continuously infused with 2 ml/kg/h crystalloid. When SVV > 12%, a bolus of 3 ~ 5 ml/kg polygelatin was administered within 10 min and then repeatedly until SVV ≤ 12%. The perioperative patient blood management followed the Transfusion of Whole Blood and Blood Components WS/T 623–2018 guidelines of China. When haemoglobin was lower than 80 g/L, a red blood cell suspension was infused to maintain haemoglobin ≥ 80 g/L. During surgery, blood products and factor concentrates were transfused according to blood loss and coagulation function tests. Autologous blood reinfusion technology was used when necessary. In the anhepatic phase, hemodynamic instability was anticipated and dominantly treated by vasopressors to maintain a mean arterial pressure (MAP) greater than 60 mmHg. If necessary, patients were treated with continuous intravenous dopamine administration (3 ~ 10 µg/kg/min) and intermittent intravenous norepinephrine administration to maintain stable circulation. 5% Sodium bicarbonate was infused to maintain acid-base balance according to the results of arterial blood gas analysis. If BE was <-4 mmol/L, 5% sodium bicarbonate (mL) was infused according to the formula 1/2 ~ 2/3 [BE× weight (kg) × 0.25 × 1.6]. Appropriate calcium chloride and potassium chloride were infused to maintain electrolyte balance.

All patients underwent conventional OLT without venovenous bypass. 1000 ml crystalloids were used to prepare the 2% albumin solution which was used for flushing before graft reperfusion. The perfusion solution was inflowed from the anastomosis of the suprahepatic and infrahepatic inferior vena by and outflowed the portal vein (PV) to flush the University of Wisconsin (UW) preservation solution.

After the completion of surgery, all patients were transferred to the intensive care unit (ICU) for ongoing monitoring and postoperative care.

### Intervention

The crystalloid used for background infusion during goal-directed fluid management and retrograde perfusion before reperfusion was AR or BR solution depending on the group. BR solution is a new type of crystalloid solution composed of various electrolytes including Na^+^ 130 mmol/L, K^+^ 4.0 mmol/L, Ca^2+^ 1.5 mmol/L, Mg^2+^ 1.0 mmol/L, Cl^−^ 109 mmol/L, HCO_3_^−^ 28 mmol/L and Citrate_3_^−^ 1.3 mmol/L. In our centre, AR solution includes Na^+^ 137 mmol/L, K^+^ 4.0 mmol/L, Ca^2+^ 1.65 mmol/L, Mg^2+^ 1.25 mmol/L, Cl^−^ 110 mmol/L, and acetate^−^ 36.8 mmol/L (Table 1).

**Table 1:** Characteristics of Bicarbonate Ringer’s solution and Acetate Ringer’s solution

	Acetate Ringer’s solution (mmol/L)	Bicarbonate Ringer’s solution (mmol/L)
Na^+^	137	130
K^+^	4.0	4.0
Ca^2+^	1.65	1.5
Mg^2+^	1.25	1.0
Cl^−^	110	109
HCO_3_^−^	0.0	28
Citrate_3_^−^	0.0	1.3
acetate^−^	36.8	0.0

### Data collection

The time of the anhepatic phase, the time of surgery, blood loss, and the amount of transfused blood were recorded. The anhepatic phase began with the clamping of the IVC and ended with the opening of the IVC and PV. Transfusion volume is defined as the total volume of red blood cells, plasma, clotting factors, and transfused platelets. A decrease in the MAP ≥ 30% within 5 min, regardless of whether vasoactive drugs were used after PV reperfusion, was defined as postreperfusion syndrome (PRS). The total amount of 5% sodium bicarbonate infused during surgery was recorded. Arterial blood gas was collected and recorded before the start of surgery (T0), during anhepatic phase (T1), immediately after reperfusion of the transplanted liver (T2), and 30 min after reperfusion of the transplanted liver (T3).

### Outcomes

The primary outcomes were arterial pH, BE, PCO_2_, blood glucose and lactate concentration at T1, T2 and T3. The secondary outcomes included the amount of 5% sodium bicarbonate infusion required during surgery and the number of patients requiring it, incidence of PRS, lengths of postoperative ICU and hospital stay, bilirubin, aspartate transaminase (AST), γ-GT, INR, and creatinine at 7 and 30 days postoperatively, and the rate of RRT.

### Statistical analysis

The data were analysed using IBM SPSS Statistics for Windows version 23.0. We followed the modified intention-to-treat analysis; only subjects who were randomized and who received all of the study interventions were included in the final analysis.

Data are expressed as the means and standard deviations (SDs) for normally distributed continuous variables and medians and interquartile ranges (IQRs) for nonnormally distributed continuous variables. Categorical variables were reported as frequencies and proportions. Normality testing was performed using the Shapiro‒Wilk test. Student’s t test was used for comparison of normally distributed variables. The Mann‒Whitney U test was used for comparison of nonnormally distributed variables between the two groups. Categorical variables were analysed via the Pearson χ2 test or the Fisher’s exact test as appropriate. All tests were 2-tailed, and *P* < 0.05 was considered statistically significant.

## Results

**Fig. 1:**
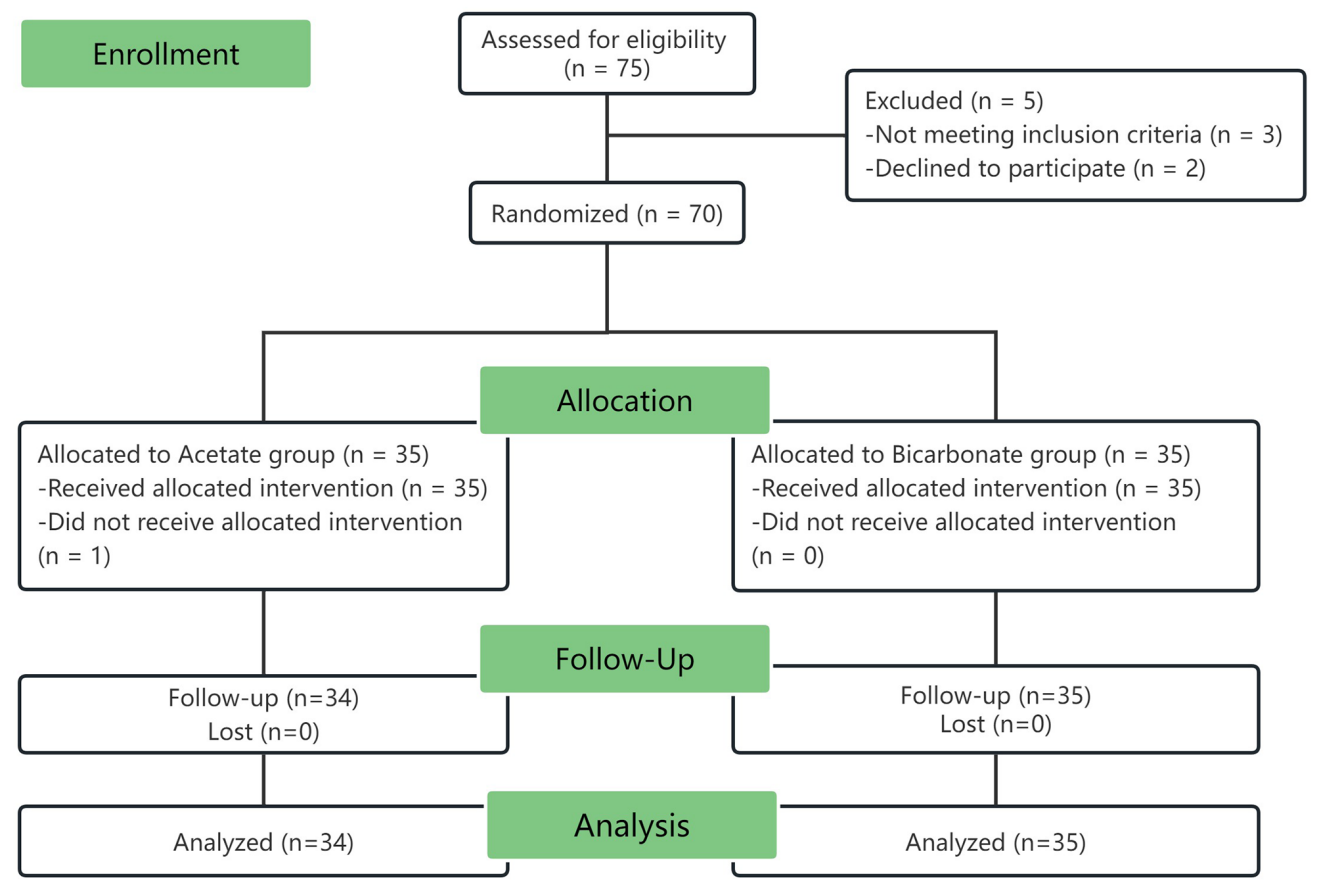
Study Flow-Chart

A total of 75 patients in the study were assessed for eligibility, of whom 3 met the exclusion criteria and 2 refused to consent to enrolment. The remaining 70 patients were randomized (35 in each group) and analysed. One patient in the acetate group was discontinued due to major bleeding during intraoperative liver resection, and there were no missing data. The study finally included 69 patients. There were 34 patients in the acetate group and 35 patients in the bicarbonate group for analysis (Fig. 1). The baseline characteristics of the patients were similar between the two groups (Table 2). Most patients were male (69.6%), and the median age was 59 (38–74) years. There were no significant differences in anhepatic stage time, operation time, blood loss volume or blood transfusion volume between the two groups, and brain death in the source of the donor liver applied for 76.8% of the two groups.

**Table 2:** Baseline characteristics of the patients

		Acetate group (n = 34)	Bicarbonate group (n = 35)
Age (years)		58 (38–70)	60 (40–74)
Females/Males		11/23	10/25
BMI (kg/m^2^) ^c^		25.1 (23.4–27.0)	25.1 (22.6–28.6)
MELD		13 (10–18)	14 (9–18)
Abdominal surgery history ^a^		9 (26)	9 (26)
**Preoperative diagnosis** ^a^	Hepatic cell carcinoma	13 (38)	13 (37)
	HBV Cirrhosis	14 (41)	15 (43)
	Others	7 (21)	7 (20)
Anhepatic phase duration (min) ^b^		40.1 ± 3.8	36.3 ± 3.6
Operating time (min) ^b^		380 ± 108	400 ± 98
Transfusion volume (mL)		1200 (800–1350)	1150 (600–1300)
Blood loss (mL)		2437 (620–3973)	2245 (750–3245)
**Donor features**	Age (years)	55 (44–66)	55 (46–65)
	Females/Males	14/20	15/20
	BMI (kg/m^2^)	26.3 (23.7–30.5)	27.0 (23.7–30.6)
	Brain death ^a^	26 (76)	27 (77)
	Heart death ^a^	8 (24)	8 (23)

Values are represented as the median (IQR) unless indicated otherwise

^a^ Values in parentheses are percentages

^b^ Data are represented as the mean ± SD. SD, standard deviation

^c^ BMI = body mass index

**Fig. 2:**
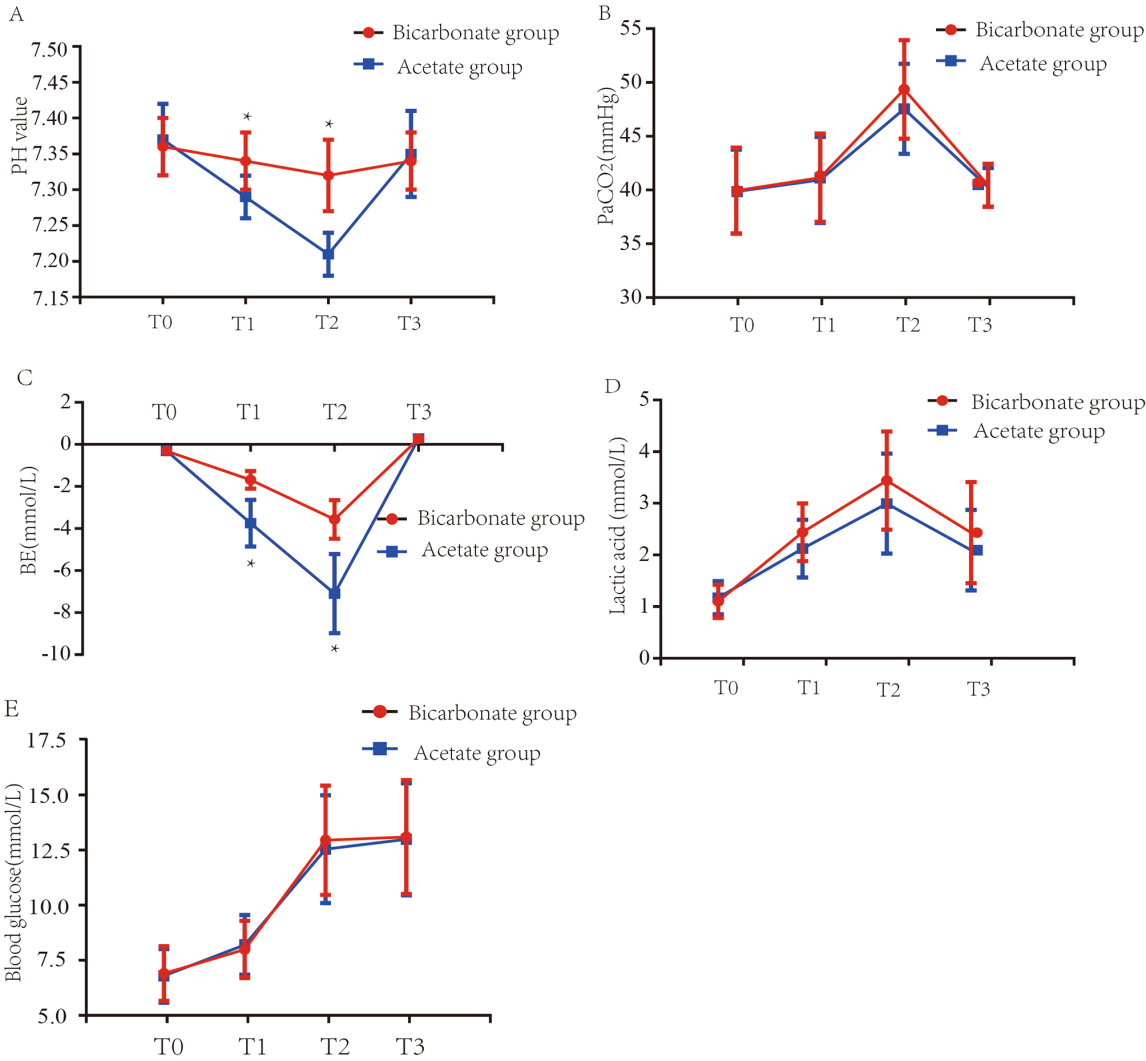
Serial changes in the PH (**A**), PaCO_2_ (**B**), BE (**C**), Lactic acid (**D**) and Blood glucose (**E**) levels before the start of surgery (T0), during the anhepatic phase (T1), immediately after reperfusion of the transplanted liver (T2) and 30 min after reperfusion of the transplanted liver (T3). *Statistically significant difference between the two groups. Data are represented as mean ± SD. SD, standard deviation

Blood glucose and lactate levels, pH, CO_2_ pressure and BE at different time points were compared between the two groups. The pH value was significantly higher in the bicarbonate group than in the acetate group at T1 (7.29 ± 0.03 vs. 7.34 ± 0.04, *P* = 0.007); the absolute BE value was significantly lower in the bicarbonate group than in the acetate group at T1 (-3.75 ± 1.12 vs. -1.68 ± 0.43, *P* < 0.001). The pH value was significantly higher in the bicarbonate group than in the acetate group at T2 (7.21 ± 0.03 vs. 7.32 ± 0.05, *P* < 0.001); the absolute BE value was significantly lower in the bicarbonate group than in the acetate group at T2 (-7.10 ± 1.89 vs. -3.57 ± 0.92, *P* < 0.001). There were no significant differences between the two groups in CO_2_ pressure or lactate or glucose levels (all *P* > 0.05). See Table 3; Fig. 2.

**Table 3:** Intraoperative arterial blood gas analysis

	Acetate group (n = 34)	Bicarbonate group (n = 35)	*P* value
Blood PH value			
T0	7.37 ± 0.05	7.36 ± 0.04	0.243
T1	7.29 ± 0.03	7.34 ± 0.04	0.007
T2	7.21 ± 0.03	7.32 ± 0.05	< 0.001
T3	7.35 ± 0.06	7.34 ± 0.04	0.316
Blood CO_2_ pressure			
T0	40.0 ± 3.9	40.1 ± 4.0	0.832
T1	41.1 ± 4.0	41.3 ± 4.1	0.235
T2	47.7 ± 4.2	49.5 ± 4.6	0.095
T3	40.4 ± 1.8	40.6 ± 2.0	0.832
BE			
T0	-0.30 ± 0.12	-0.32 ± 0.19	0.604
T1	-3.75 ± 1.12	-1.68 ± 0.43	< 0.001
T2	-7.10 ± 1.89	-3.57 ± 0.92	< 0.001
T3	0.29 ± 0.18	0.28 ± 0.17	0.109
Blood lactate			
T0	1.08 ± 0.32	1.01 ± 0.32	0.688
T1	2.03 ± 0.56	2.35 ± 0.56	0.172
T2	2.90 ± 0.97	3.35 ± 0.95	0.056
T3	2.00 ± 0.78	2.34 ± 0.98	0.169
Blood glucose			
T0	6.60 ± 1.22	6.70 ± 1.24	0.566
T1	8.00 ± 1.36	7.80 ± 1.31	0.338
T2	12.35 ± 2.45	12.75 ± 2.48	0.157
T3	12.80 ± 2.55	12.90 ± 2.58	0.761

Data are represented as the mean ± SD. SD, standard deviation

T0 = before the start of surgery; T1 = anhepatic phase; T2 = immediately after reperfusion of the transplanted liver; T3 = 30 min after reperfusion of the transplanted liver

The number of patients who needed an infusion of 5% sodium bicarbonate was 32 (94%) in the acetate group and 27 (77%) in the bicarbonate group (*P* = 0.045). The median (25-75th percentiles) of the 5% sodium bicarbonate volume infused during the operation in the acetate group and bicarbonate group was 175 (50–280) and 110 (50–200), respectively (*P* = 0.0000). The numbers of patients who experienced PRS in the acetate group and bicarbonate group were 16 (47%) and 10 (29%), respectively (*P* = 0.113) (Table 4).

**Table 4:** Other intraoperative data

	Acetate group (n = 34)	Bicarbonate group (n = 35)	*P* value
Incidence of 5% sodium bicarbonate infusions	32 (94)	27 (77)	0.045
Volume of 5% sodium bicarbonate infusions (ml) ^a^	175 (50–280)	110 (50–200)	0.0000
RPS	16 (47)	10 (29)	0.113

Values in parentheses are percentages unless indicated otherwise

^a^ Values are the median (IQR)

Primary postoperative outcomes are shown in Table 5. There was no significant difference in ICU stay or hospital stay between groups. The AST levels at 7 postoperative days in the acetate and bicarbonate groups were 318.5 ± 176.8 IU/L and 247.5 ± 162.5 IU/L, respectively, with significant differences (*P* = 0.0000). The creatinine level at 30 postoperative days in the two groups was 90.2 ± 27.2 µmol/L and 82.2 ± 16.3 µmol/L, respectively (*P* = 0.019). There were no significant differences in AST over 30 days postoperatively, creatinine over 7 days postoperatively, bilirubin, γ-GT and INR over 7 and 30 days postoperatively, or the number of patients requiring RRT.

**Table 5:** Postoperative outcomes

	Acetate group (n = 34)	Bicarbonate group (n = 35)	*P* value
ICU discharge ^a^	4 (3–7)	4 (2–7)	0.339
Hospital discharge ^a^	15 (11–24)	15 (10–24)	0.926
AST (IU/L)			
postoperative day 7	318.5 ± 176.8	247.5 ± 162.5	0.0000
postoperative day 30	23.4 ± 15.5	21 ± 12.8	0.707
Bilirubin (µmol/L)			
postoperative day 7	48.1 ± 23.4	47.5 ± 22.7	0.229
postoperative day 30	14.0 ± 7.5	14.0 ± 7.4	0.479
γ-GT (IU/L)			
postoperative day 7	302.2 ± 102.4	268.1 ± 87.9	0.157
postoperative day 30	200 ± 69.0	178 ± 58.4	0.949
INR			
postoperative day 7	1.2 ± 0.2	1.2 ± 0.2	0.644
postoperative day 30	1.1 ± 0.1	1.1 ± 0.1	0.735
Creatinine (µmol/L)			
postoperative day 7	97.2 ± 27.2	92.8 ± 20.1	0.139
postoperative day 30	90.2 ± 22.5	82.2 ± 16.3	0.019
Renal replacement therapy			
postoperative day 7 ^b^	3 (8)	3 (9)	0.970
postoperative day 30 ^b^	4 (12)	3 (9)	0.581

Data are represented as the mean ± SD, unless indicated otherwise. SD, standard deviation

^a^ Values are the median (IQR)

^b^ Values in parentheses are percentages

## Discussion

This study is the first randomized trial in which the effects of BR solution infusion in target-directed fluid therapy combined with retrograde refusion in patients undergoing liver transplantation surgery were investigated. We found that BR solution infusion improved intraoperative acid-base equilibrium, especially at time points in the anhepatic stage and immediately after reperfusion of the transplanted liver and reduced postoperative hepatocellular enzymes.

The most important finding of this study was that BR solution may perform better than AR solution in maintaining acid-base status after OLT. Normal liver function is necessary for the prevention of lactic acidosis via recapture of circulating lactate by hepatocytes and conversion of lactate to glucose via the Cori cycle [14]. The metabolism of OLT patients is slow due to liver dysfunction and the anhepatic phase [15]. AR solution is mainly metabolized through the liver and eventually converted to sodium bicarbonate; this process normally takes 15 min and can increase the burden of the new liver after transplantation [16]. In contrast, BR solution metabolism is independent of liver processes, and only 10% of BR solution is excreted through the kidney, which has little impact on liver function [17, 18]. Therefore, in our study, the pH value was significantly higher and the absolute BE value was lower in the bicarbonate group than in the acetate group at T1. At the neohepatic stage (T2), as lactic acid or unmeasured anions enter the blood circulation, metabolic acidosis is more likely to occur [14]. However, BR solution, a novel balanced crystalloid buffered with bicarbonate rather than organic anions which provides physiological levels of bicarbonate ions and electrolyte ions, can be used to supplement missing extracellular fluid and correct metabolic acidosis promptly. Therefore, the pH and absolute BE value were significantly higher in the bicarbonate group than in the acetate group at T2. Lv [9] et al. reported that BR solution was more effective regarding acid-base status than AR solution following OLT. Figiel [19] et al. demonstrated that metabolic acidosis during the reperfusion phase of LT is directly associated with impaired coagulation and dramatic haemodynamic fluctuations. A stable internal environment could be valuable for postoperative early extubation [20]. In summary, the benefits of using BR solution are obvious.

In addition to maintaining the acid-base balance, using BR solution also had advantages in reducing the volume and incidence of 5% sodium bicarbonate infusion. Due to the improved acid-base equilibrium, less volume and fewer incidents of 5% sodium bicarbonate infusion are needed. Our results showed that the number of patients who needed an infusion of 5% sodium bicarbonate was 32 (94%) in the acetate group and 27 (77%) in the bicarbonate group (*P* = 0.045). The median (25-75th percentiles) of the 5% sodium bicarbonate volume infused during the operation in the acetate group and bicarbonate group was 175 (50–280) and 110 (50–200), respectively. Previous studies showed that the incidence and volume of 5% sodium bicarbonate infusion were 91–98% and 170–350 ml, respectively, which is in line with our study [21]. In a meta-analysis that included five RCTs (1079 patients), the use of 5% sodium bicarbonate prolonged the duration of ventilation and ICU length of stay and increased the risk of alkalemia [22].

AST level is the commonly used clinical index that reflects hepatocyte injury. In this study, it was found that the 7-day AST values in the two groups were 318.5 ± 176.8 IU/L and 247.5 ± 162.5 IU/L, respectively. The difference was statistically significant, indicating that there was less liver cell injury in the bicarbonate group. The mechanism of hepatocyte injury is related to liver cryopreservation, inflammatory cytokine release and IRI [23]. Compared with AR solution, BR solution significantly reduced liver cell apoptosis induced by ischaemia‒reperfusion and reduced the release of inflammatory cytokines. Previous studies have shown that 7-day AST values were negatively correlated with long-term graft liver function and patient survival in LT. This study also found that the creatinine values of the two groups at 30 days after surgery were 90.2 ± 22.5 µmol/L and 82.2 ± 16.3 µmol/L, respectively, with a significant difference (*P* = 0.019); this result is in line with those of other reports [24]. However, since the results of this study were only for secondary outcomes and the sample size was small, their significance needs to be further verified.

Although donation after brain death (DBD) represents the first choice for organ donation, the China Organ Transplant Response System reported that donation after cardiac death (DCD) was the source of livers for approximately 40% of LTs during the 2018–2020 period [25, 26]. The beginning of the warm ischemic time (WIT) of a DBD donor is considered the time that the arterial clamp is closed, while the WIT of a DCD donor is the time from a MAP < 50 mmHg or an arterial saturation of < 80% until the start of cold perfusion [27]. Notedly, WIT correlates with IRI and postoperative systemic inflammatory response, which is more deleterious to hepatocytes and contributes to poor liver status, such as biliary necrosis, cholangitis, and graft failure [28]. Leithead [29] et al. found that DCD recipients had greater intraoperative hemodynamic instability and greater intraoperative transfusion requirements in a retrospective study. In contrast, a meta-analysis indicated that DCD grafts resulted in patient and graft survival rates similar to those of DBD grafts; in addition, both types of transplanted organs appeared to be equivalent in terms of postoperative complications and the length of hospital stay [30]. In our study, the proportion of DBD and DCD grafts in the two groups were comparable.

Limitations of the study included the small sample size and lack of comparison of HCO^3−^ levels. The data should have been collected up to the end of the operation and 24 h postoperatively. Postoperative coagulation function was not assessed or analysed. Haemodynamics were not analysed during the operation. Only a single type of surgical operation, whole LT, was considered in the study, and patients with living-related liver transplantation were not included. Although we compared the transfusion volume, blood loss, donor features and PRS in the two groups, we did not consider other confounding factors that may influence the BE and PH levels. Further research involving a larger number of patients undergoing different types of LT surgeries and with other commonly used crystalloids is needed to understand the acid-base physiology in a broad range patients.

In conclusion, this randomized controlled trial showed that compared with AR solution, infusion of BR solution could help maintain the normal intraoperative acid-base balance, especially during the anhepatic stage, as well as provide more base reserve and reduce the need for 5% sodium bicarbonate administration. In addition, the use of BR solution could decrease the AST level at 7 postoperative days and the creatinine levels at 30 postoperative days, which indicated that BR solution may potentially protect graft liver function early after surgery and reduce renal injury late after surgery. These effects can potentially improve the final prognosis. Therefore, BR solution can be recommended as an alternative to AR solution.

## Data Availability

The datasets used and/or analysed during the current study are available from the corresponding author upon reasonable request.
